# Variations in Current and Desired Limb Perception in Complex Regional Pain Syndrome

**DOI:** 10.1002/ejp.70401

**Published:** 2026-10-09

**Authors:** Caitlin E. Naylor, Gavin Buckingham, Christof Lutteroth, Janet H. Bultitude

**Affiliations:** ^1^ Department of Psychology University of Bath Bath UK; ^2^ Department of Public Health and Sport Sciences University of Exeter Exeter UK; ^3^ Department of Computer Science University of Bath Bath UK; ^4^ School of Medicine and Psychology Australian National University Canberra Australia

## Abstract

**Background:**

People with Complex Regional Pain Syndrome (CRPS) often experience body perception disturbances, whereby features of the affected limb are perceived as distorted. However, these disturbances vary widely between individuals, as does the effectiveness of treatments aimed at altering body perception. It remains unclear whether such variation reflects distinct underlying patterns of disturbances and whether individuals differ in how they would change their limb perception.

**Methods:**

We conducted an exploratory online survey to examine current and desired perceptions of the affected limb in people with CRPS. 115 people with CRPS reported the severity of visual and felt disturbances in the perceived size, shape, position and definition of their affected limb, as well as any changes they would like to make to this perception.

**Results:**

Three clusters of body perception disturbances were identified, differing in the degree of perceptual distortion from a typical limb and in the level of multimodal discrepancy. These clusters were associated with differences in the severity of other CRPS symptoms. Another three clusters were identified based on desired perceptual changes, with most respondents seeking alterations that did not simply involve returning the limb to a normal state.

**Conclusions:**

These findings challenge the common practice of treating body perception disturbance as a single symptom in CRPS. They illustrate the value of recognising how disturbances manifest and show that individuals' desired perceptual changes often diverge from traditional rehabilitation methods aimed at normalising the limb. Future work should explore tailoring rehabilitation to individual body‐perception profiles.

**Significance Statement:**

Body‐perception disturbance in CRPS is not a single construct, but comprises distinct patterns linked to clinical severity. Many individuals do not wish their limb to simply be normal, challenging core rehabilitation assumptions. These insights demonstrate substantial heterogeneity in how people with CRPS perceive their limb and how they want it to be. This highlights the need for personalised, patient‐centred approaches to body‐perception–focused pain management in CRPS.

## 1 Introduction

Complex regional pain syndrome (CRPS) is a chronic condition affecting one or more limbs (Goh et al. 2017). Besides pain, 70%–90% of people with CRPS experience distorted limb perceptions (Halicka et al. 2020; Lewis et al. 2007; Ten Brink et al. 2020). These body‐perception disturbances are associated with pain and sensorimotor impairments (Bultitude and Rafal 2010; Lewis and Schweinhardt 2012; Schulte‐Goecking et al. 2022) and may contribute to the maintenance of CRPS (Büntjen et al. 2017; Halicka et al. 2020; Lotze and Moseley 2007; Tsay et al. 2015). Although previous research describes complex disturbances across multiple dimensions of body perception, the extent or importance of this variability has not been systematically investigated. This study aims to characterise body‐perception disturbances in CRPS and their relationship to CRPS symptoms and function.

Research in pain‐free participants suggests that the brain maintains multiple body representations that can be independently shaped by visual or somatosensory information (Longo 2015). In CRPS, body‐perception disturbances can affect the limb's visual appearance (e.g., looks larger than actual size), feeling (e.g., feels displaced relative to physical location), and mental representation (e.g., impression in ‘mind's eye’ is missing fingers; Förderreuther et al. 2004; Lewis et al. 2007; Lewis and McCabe 2010; Lewis and Schweinhardt 2012; McCabe et al. 2005). Moreover, disturbances can extend beyond specific limb features to broader bodily experiences, including altered bodily continuity, ownership and agency. However, because body‐perception disturbances are typically characterised as a single construct, it is uncertain whether their heterogeneity reflects meaningful differences in CRPS. It also remains unclear whether disturbances manifest as differences from a typical limb, or as conflicts between sensory modalities, or both. Characterising disturbance variation is a necessary first step to determine whether specific disturbance patterns reflect distinct mechanisms, clinical profiles or treatment needs.

Given the heterogeneity of body‐perception disturbances in CRPS, understanding what individual patients perceive as desirable may help identify more appropriate treatment targets. Some interventions aim to correct disturbances by normalising the limb's appearance, such as mirror box therapy (McCabe et al. 2008; Sayegh et al. 2013), although effectiveness is mixed (Boesch et al. 2016). Virtual reality is increasingly used to recalibrate body representation in chronic pain (Maggio et al. 2025); for instance, Matamala‐Gomez et al. (2019) altered the size of an embodied arm in virtual reality and reported analgesia when it appeared smaller than normal, but not larger. However, these manipulations defined size relative to a typical limb, not accounting for patients' existing (mis‐)perception of limb size. This reflects a limitation common to such approaches (Boesch et al. 2016; Maggio et al. 2025), which typically apply the same manipulation across patients, despite heterogeneity in perceptual disturbances. Lewis et al. (2021) provide preliminary support for individualised treatments, showing that matching a virtual hand to each individual's desired appearance reduced pain and body‐perception disturbances. Consequently, understanding desired limb perception may help identify what perceptual changes patients believe would be needed to restore a coherent body representation for individual patients.

Given the lack of systematic characterisation of body‐perception disturbances in CRPS and uncertainty regarding which dimensions of perceptual variability are clinically meaningful, we conducted an exploratory survey of body‐perception experiences in CRPS. Our primary aim was to characterise variability in these disturbances and understand how people with CRPS desire their affected limb to be. The secondary aim was to examine the practical importance of individual differences in body‐perception disturbances, specifically their relationship with CRPS severity, pain and daily functioning.

## 2 Method

### 2.1 Survey

#### 2.1.1 Survey Distribution

Ethical approval was obtained from the University of Bath's Psychology Research Ethics Committee (code 21‐272). We distributed the survey via an anonymous link shared on social media, health charity mailing lists, chronic pain blogs, and via email to individuals who had consented to be contacted about CRPS research opportunities. We recruited 164 adults with chronic limb pain, defined as pain in one or more limb(s) on most days for 3 months or more, between February and June 2022. To minimise burden, respondents could end the survey after completing the ‘desired limb perception’ section, which provided sufficient data for the primary aims; remaining questions addressing secondary aims were optional. Respondents could take breaks at any time and had 1 week to complete the survey. On completion, they could enter a prize draw for a £50 Amazon voucher (or local currency equivalent).

We created the survey using Qualtrics (available here). Two individuals with CRPS completed the survey prior to launch and provided verbal feedback on question wording, meaning and focus. The final survey included the following sections (detailed in Sections 2.1.2, 2.1.6): demographics and pain characteristics; current perception of the affected limb; desired changes to the affected limb; impact on functioning and pain. Additional qualitative questions were included but will be reported separately due to the survey's large scope.

#### 2.1.2 Participant Demographics and Pain Condition

Respondents provided demographic information (age, gender, and handedness). They also provided details of their chronic pain experience, including diagnosis, duration, affected limbs and their most painful limb for cases where multiple body areas were affected. They also completed the Short Form Brief Pain Inventory (BPI; Cleeland 2009).

#### 2.1.3 Current Perception of the Affected Limb

This section began with the revised Bath CRPS Body Perception Disturbance Scale (r‐B‐CRPS‐BPD; Ten Brink et al. 2020). Respondents were then asked about how their affected limb looked to them, referring to its perceived visual appearance, and how it felt to them, referring broadly to feelings, impressions or mental images of the limb. They rated how their affected limb *looked* and *felt* compared with a typical, healthy, or unaffected limb, across four features: size, shape, position and definition. Definition was described as the distinctness of the limb's outline, including the sharpness of its boundaries and the spaces between the fingers. Each rating was provided on a sliding scale of 0–100, where 0 represented no difference and 100 represented significant differences. Given the exploratory nature of our study and the fact that there is no clear threshold at which limb perception could be considered disturbed, we considered any response over 10 as indicating distorted limb perception.

To understand whether respondents believed any reported changes to the visual appearance of the limb were due to physical alterations (e.g., swelling) or a perceptual disturbance (e.g., perceiving the limb to appear larger than its true size), we asked them to indicate whether they believed their perceived visual limb appearance matched the limb's physical state.

#### 2.1.4 Desired Changes to the Affected Limb

This section asked if respondents would want to change how they perceived their limb's visual appearance or feeling, and whether they desired their limb to resemble a typical limb. Respondents rated the extent to which they would want to change how they perceived their limb's visual appearance and feeling, across the four features of size, shape, position and definition (0 = no change—100 = extreme changes). If respondents gave a rating of higher than 10, they rated the extent to which the new visual appearance and feeling of their affected limb would compare to that of a typical, healthy, or unaffected limb (0 = same as a typical limb—100 = extremely different to a typical limb, that is, atypical).

#### 2.1.5 Impact on Functioning

Respondents reported whether the limb's appearance or feeling negatively affected their satisfaction with their limb, general daily functioning or specific daily activities (grasping a mug, lifting a cup, writing, typing, brushing teeth, walking up stairs, walking for 10 min, getting dressed, showering/bathing, preparing food, tying shoe laces, and bending to pick something up). These activities were selected because they are relevant to CRPS presentation and are included in standard assessments of limb function (e.g., Disabilities of the Arm, Shoulder and Hand questionnaire, Gummesson et al. 2003; Lower Extremity Functional Scale, Binkley et al. 1999; Upper Limb Functional Index, Gabel et al. 2006). If respondents reported both functional impacts and had expressed a desire to change their limb perception, they then rated the extent to which achieving their desired limb perception would improve their functional experiences (0 = no expected improvement—100 = expected extreme improvement).

#### 2.1.6 General Impact of Pain

In this final section, respondents indicated which symptoms from the CRPS Severity Scale they experienced (Harden et al. 2010, 2017) and completed the Interference section of the Short Form Brief Pain Inventory (Cleeland 2009). They then reported whether they had any mobility difficulties, and if so, they rated the degree to which their mobility was reduced and interfered with everyday life (0 = very little–100 = extreme reduction/interference).

### 2.2 Exclusions and Demographics

Data from partially completed surveys were included in analyses, except for eight respondents who were excluded because they dropped out of the survey before answering any questions regarding body perception. We removed a further 41 people who experienced chronic pain but were not diagnosed with CRPS, as this non‐CRPS sample was too small for a valid comparative analysis and was not essential to our primary aims. All data, including the non‐CRPS sample, are available here.

The final sample consisted of 115 individuals with CRPS (1 non‐binary person; 14 men; 100 women) aged 16–78 years (*Med* = 49, IQR = 20). This gender imbalance is consistent with CRPS being more frequently reported in females than males (de Mos et al. 2007), for instance, in 2015, the UK CRPS registry consisted of 72.5% females (Shenker et al. 2015). CRPS affected the left upper limb in 17%, the right upper limb in 21%, the left lower limb in 33%, and the right lower limb in 29% of respondents. CRPS duration ranged from 5 months to 34 years (Mdn = 8.5, IQR = 8). Eighty‐eight respondents reported pain in multiple body parts, though survey items always referred to their most painful limb.

## 3 Results

Given the exploratory nature of this investigation, we justify and report the analyses used throughout the results section. As Shaprio–Wilk tests indicated non‐normality (*W*s > 0.82, *p*s < 0.001), we report medians and interquartile ranges. As we did not find outliers in the c*urrent body‐perception* scores (questions detailed in Section 2.1.3) or desired *body‐perception* scores (questions detailed in Section 2.1.4), the use of Pearson's correlations (Bonferroni corrected) was appropriate despite non‐normal data (Chok 2010). We report the number of participants (*n*) that answered each set of questions, as participants dropped out throughout the survey. Any composite indices computed from the questions we created for the survey or clustered group names will be italicised for clarity. All data and analysis outputs are available here.

### 3.1 Current Perception of Affected Limb

#### 3.1.1 Summary of Reported Perceptual Disturbances

Most respondents (96% of *n* = 115) reported at least one distortion to the visual appearance or feeling of their affected limb (defined as differences between perception of limb and a typical limb above 10/100). Of the 92 respondents who reported visual disturbances and answered whether perceived limb appearance matched the physical state, 38% indicated it did not match the physical state, 48% reported a match and 14% were unsure.

Across the ‘current body perception’ questions, most respondents reported visual distortions in size (84% of *n* = 114), shape (86% of *n* = 111), position (77% of *n* = 110), or definition (70% of *n* = 105), with 61% of respondents (of *n* = 105) reporting visual distortions across all features. Similarly, most respondents reported distortions in their feeling of limb size (88% of *n* = 95), shape (88% of *n* = 93), position (76% of *n* = 93), or definition (73% of *n* = 92), with 63% of respondents (of *n* = 92) reporting felt distortions across all features. Median ratings ranged from 50 to 69 (Figure 1), indicating notable disturbances across all features. However, responses spanned the entire scale for all features, reflecting a high degree of heterogeneity in disturbance severity.

**FIGURE 1:**
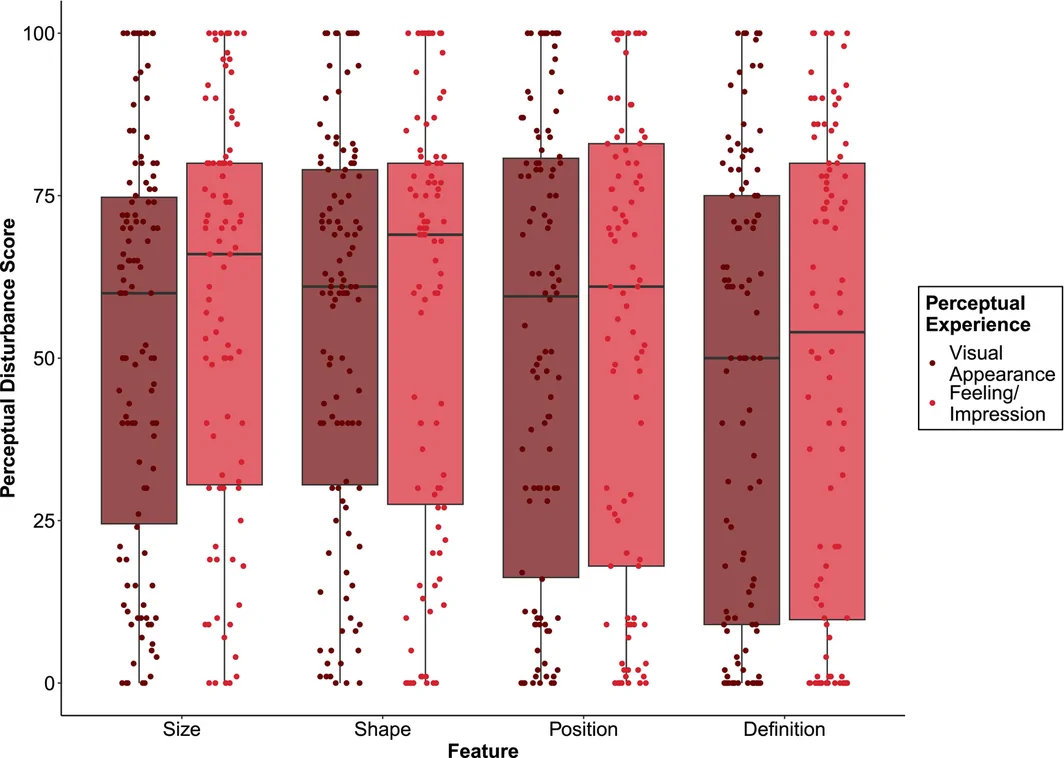
Extent of perceptual disturbances in CRPS impacting visual appearance and feeling of the affected limb across different features. The horizontal black line represents the median, the lower and upper hinges correspond to the first and third quartiles (the 25th and 75th percentiles), and the whiskers represent 1.5 IQR from the hinges. Dots represent individual participant data. Perceptual disturbance score for each modality represents the magnitude of disturbed limb perception.

Pearson's correlations showed that distortions across all features of visual appearance and feeling were strongly correlated (*r*s = 0.58–0.81, *p*s < 0.001; see Figure 2), indicating that different features of body perception are related to one another. To summarise overall disturbance severity in each modality, we averaged ratings across the four features to create a *Visual Disturbance Index* (*n* = 92, Mdn = 53.75, IQR = 49.75) and a *Felt Disturbance Index* (*n* = 105, Mdn = 62.5, IQR = 48.81). Despite these strong associations, the widespread of individual scores suggested that group‐level correlations could obscure individual differences between visual and felt perceptions. To explore this, we calculated a multimodal discrepancy score for each feature (size, shape, position and definition) by taking the absolute difference between individuals' visual appearance and feeling scores (see Figure 3), and then averaged these values to create a *Multimodal Discrepancy Index*. This index reflects the difference in the magnitude of visual and felt distortions, without indicating the direction of change (i.e., an increase or decrease in size). As expected from the high correlations, the median *Multimodal Discrepancy Index* was low (*n* = 93, Mdn = 10, IQR = 13); however, the full range (0–73.25) showed that some individuals experienced substantial differences between how their limb looked and felt (Figure 3).

**FIGURE 2:**
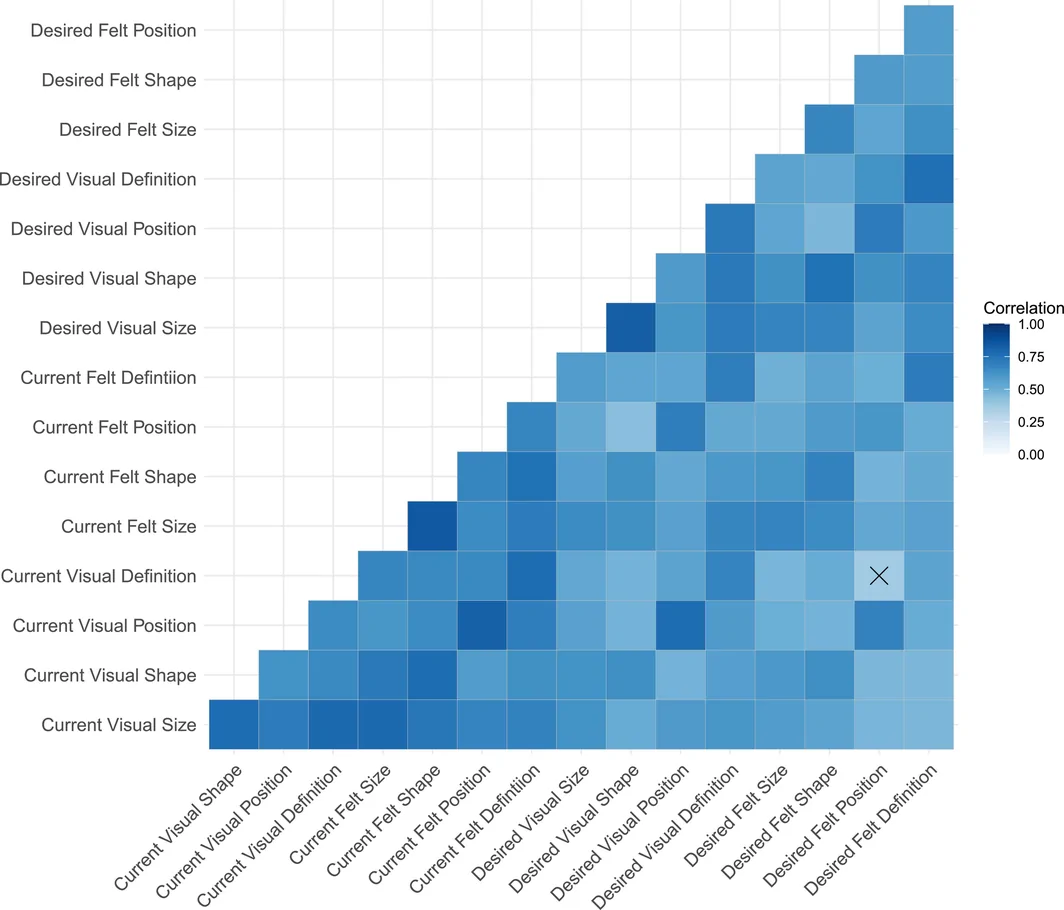
Correlation matrix to show associations between current disturbances to visual and felt limb size, shape, position and definition; and desired changes to the same features. ‘Current’ refers to the reported magnitude of disturbed limb perception. ‘Desired’ refers to the reported changes that people would make to the affected limb, if it was possible. × indicates non‐significant Bonferroni‐corrected *p*‐value.

**FIGURE 3:**
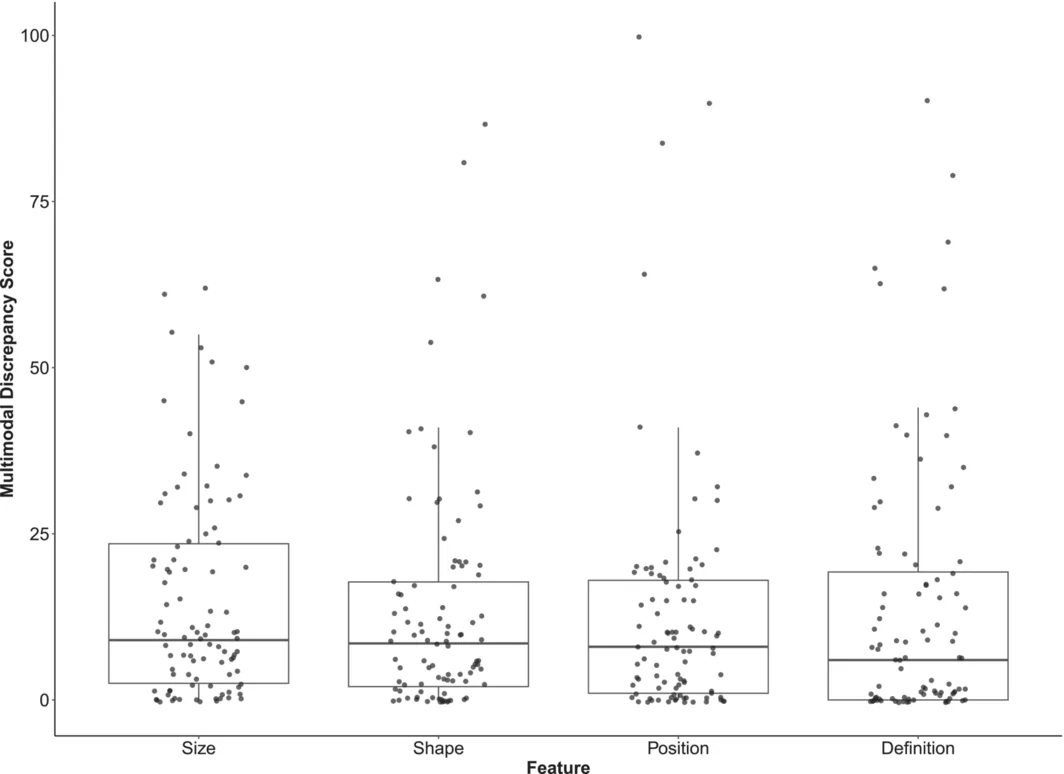
Extent of multimodal discrepancy between visual and felt body perception disturbances across different features. The horizontal black line represents the median, the lower and upper hinges correspond to the first and third quartiles (the 25th and 75th percentiles), and the whiskers represent 1.5 IQR from the hinges. Dots represent individual participant data.

#### 3.1.2 Identifying Sub‐Groups Based on Disturbances

Overall, body‐perception disturbances across different modalities are highly related, but show variability within individuals. Given this, we explored whether variability in disturbances across different modalities represented meaningful patterns for symptom profiles. We conducted an agglomerative cluster analysis (*n* = 92) to determine how participants could be grouped by their *Visual Disturbance Index*, *Felt Disturbance Index and Multimodal Discrepancy Index*. Although the *Visual Disturbance Index* and *Felt Disturbance Index* were strongly correlated (*n* = 92, *r* = 0.88, *p* < 0.001), including them separately in the cluster analysis enabled us to examine whether increased severity in one modality over the other contributed to cluster differentiation—an effect that would be obscured if they were combined into a single score. Using these two indices alongside the *Multimodal Discrepancy Index* allowed us to capture the influence of (i) variation in disturbance severity, (ii) whether severity was higher in visual or felt disturbances and (iii) the extent of multimodal disagreement.

Ward's agglomerative clustering with Euclidian distances was used to minimise within‐cluster variance. Examination of coefficients on a scree plot, along with dissection of the longest horizontal line on the dendogram, indicated a three‐cluster solution (analysis and graph interpretations available here). The first and largest cluster (58%) represents people with severe visual and felt perceptual disturbances that similarly affect both the visual appearance and feeling of their limb (named ‘Severe Disturbance with Multimodal Agreement’). The second cluster (32%) represents people with minimal disturbances across both modalities (named ‘*Minimal Disturbances*’). The third cluster (10%) represents people with pronounced multimodal disagreement, with most individuals (8/9) in the group showing more severe disturbances in the feeling of their limb compared with the visual appearance (named ‘Disturbances with Multimodal Disagreement’).

Our first two clusters, consisting of most of our respondents, were defined by the similarity of the *Visual* and *Felt Disturbance Indexes* of the people in those groups. To summarise overall disturbance severity independently of modality, we conducted an exploratory principal axis factor analysis on the eight individual disturbance items. Principal axis factoring extraction with oblimin rotation (Table S1) showed that a single factor captured the majority of variance (70.63%), with a high Eigenvalue (5.65) and high Kaiser–Meyer–Olkin measure of sampling adequacy (KMO = 0.89). This supported an overall index of perceptual disturbance severity, so we averaged answers across the 8 items for each individual and averaged these scores to compute an overall *Perceptual Disturbance Index* (*n* = 92, Mdn = 57.31, IQR = 47.28).

To characterise the cluster profiles, we compared each cluster group according to their *Perceptual Disturbance Index* and *Multimodal Discrepancy Index*. These trends support the descriptions we gave to the identified clustered groups (Figure 4). The cluster profiles indicate that individuals meaningfully vary both in overall disturbance severity and the level of multimodal agreement in severity.

**FIGURE 4:**
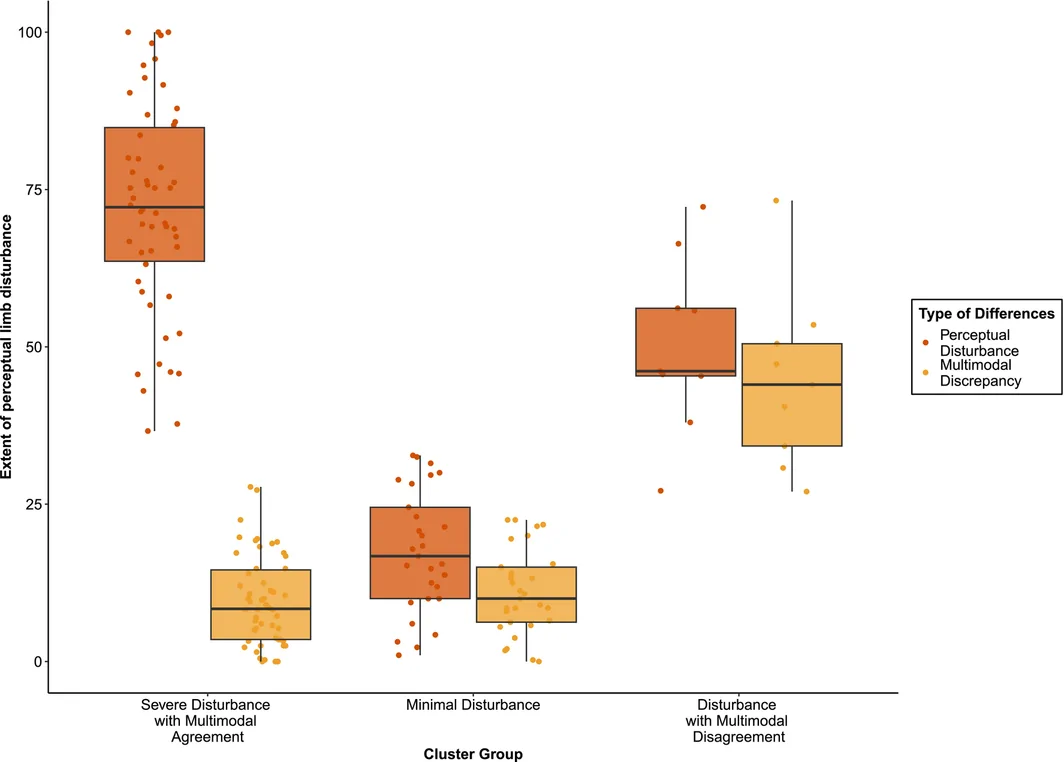
Differences in types of body perception disturbances in CRPS. The horizontal black line represents the median, the lower and upper hinges correspond to the first and third quartiles (the 25th and 75th percentiles), and the whiskers represent 1.5 IQR from the hinges. Dots represent individual participant data.

### 3.2 Desired Perception of Affected Limb

#### 3.2.1 Summary of Desired Changes to the Affected Limb

Most respondents indicated they would alter how their affected limb looked (92% of *n* = 84) or felt (93% of *n* = 75) if they could. Across the *desired body‐perception* questions, most respondents desired changes to the visual appearance of their affected limb's size (81% of *n* = 84), shape (82% of *n* = 82), position (79% of *n* = 81) or definition (69% of *n* = 78), with 62% reporting a desire to change the visual appearance of all four features. Similarly, most respondents desired changes to the feeling of their affected limb's size (88% of *n* = 75), shape (79% of *n* = 72), position (69% of *n* = 71) or definition (63% of *n* = 70), with 50% reporting a desire to change how the limb felt across all four features. Median ratings ranged from 50.5 to 74.5 (Figure 5), reflecting the common desire for some change. However, responses spanned the entire scale for all features, reflecting a high degree of heterogeneity in the extent to which people would change their affected limb.

**FIGURE 5:**
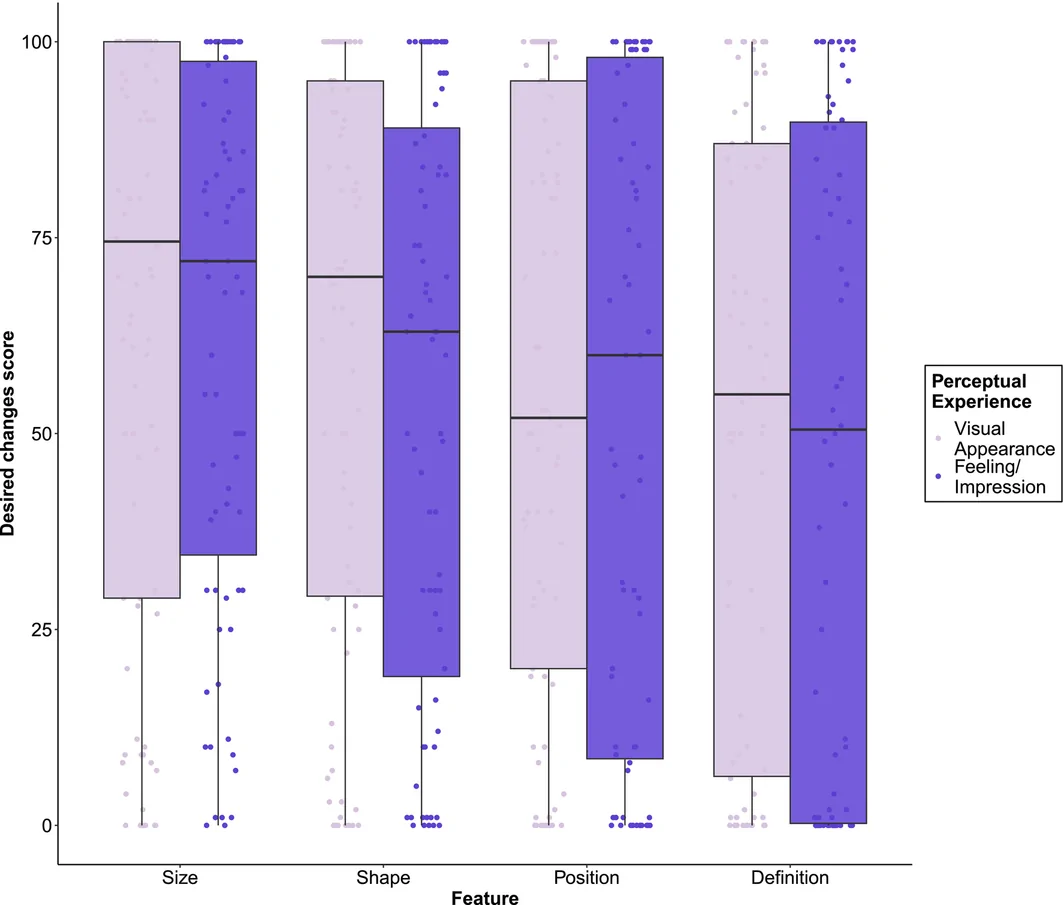
Extent that people with CRPS desire to change the visual appearance and feeling of the affected limb across different features. The horizontal black line represents the median, the lower and upper hinges correspond to the first and third quartiles (the 25th and 75th percentiles), and the whiskers represent 1.5 IQR from the hinges. Dots represent individual participant data.

Respondents also rated how their desired limb would compare to a typical limb, showing wide variability in the extent to which they desired typicality (Figure 6). Median scores (43–51.5) indicated that most people did *not* want their limbs fully restored to a typical appearance or feeling, but the wide spread of data highlights the individual variability in how perceptual disturbances would be resolved. Only 8% (*n* = 64) desired a completely normal visual appearance (scoring 0 on all features), and 10% (*n* = 62) desired their limbs to feel completely normal. Categorical responses, however, suggested that 73% of participants believed their desired limb would look typical, whereas only 21% believed it would feel typical (Table S11). The apparent discrepancy between scale and categorical responses likely reflects the widespread of scale responses: many participants may have desired a limb closer to typical, but not entirely typical, a nuance that the restrictive nature of categorical questions could not capture. As scale questions allowed respondents to express these graded preferences and nuances, we used the scale responses in the remaining analysis.

**FIGURE 6:**
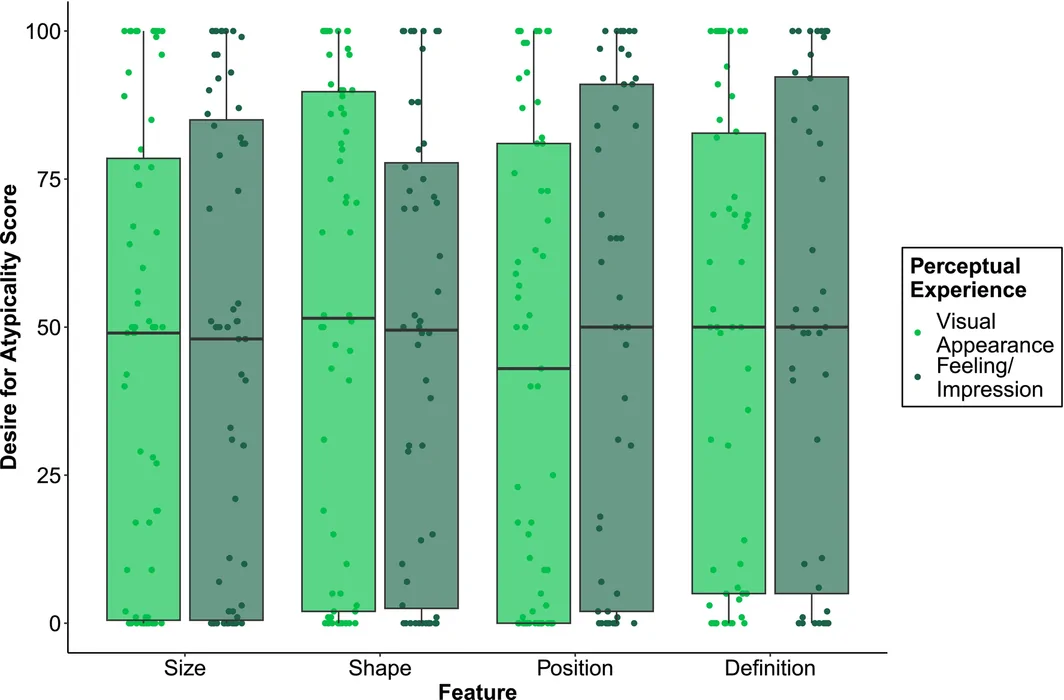
Extent to which the desired limb is different from a typical limb. The horizontal black line represents the median, the lower and upper hinges correspond to the first and third quartiles (the 25th and 75th percentiles) and the whiskers represent 1.5 IQR from the hinges. Dots represent individual participant data.

Desired changes across features were strongly correlated for both visual and felt changes (*r*s = 0.46–0.82, *p*s = 0.002 to < 0.001; See Figure 2). Accordingly, we averaged desired‐change ratings across the four features to create a *Visual Desires Index* (Mdn = 64, IQR = 56.06) and a *Felt Desires Index* (Mdn = 49.88, IQR = 55.31), representing the overall degree to which people wished to alter the appearance or feeling of their limb. Desired changes were also correlated with current perceptual disturbances (*r*s = 0.46–0.77, *p*s = 0.005 to < 0.001)—except for perceived visual definition and desired felt position (*r* = 0.37, *p* = 0.109)—indicating that the extent to which individuals wanted their limb to change aligned with the severity of their existing disturbances (Figure 2).

We also found that ratings of how different the desired limb would be from a typical limb were highly correlated across features and modalities (*r*s = 0.69–0.98, *p*s < 0.001), so we calculated a *Visual Desire for Atypicality Index* (Mdn = 50, IQR = 69.5) and a *Felt Desire for Atypicality Index* (Mdn = 49.63, IQR = 64.56). These indices capture the extent to which participants' desired limb would be different from a typical limb (i.e., high score indicates atypicality).

#### 3.2.2 Identifying Sub‐Groups Depending on Desired Changes

Given the high correlations but substantial variability in desired changes, we explore whether variability in desired changes represented meaningful patterns within individuals with CRPS. We conducted an agglomerative cluster analysis (*n* = 59) to determine how individuals could be grouped by their *Visual Desires Index, Visual Desire for Atypicality Index*, *Felt Desires Index* and *Felt Desire for Atypicality Index*. Although the four variables were correlated with one another (*r*s = 0.41–0.90, *p*s = < 0.001), using separate indices for each modality allowed us to consider any patterns in strength of desired changes or atypicality that differentiated by modality. For instance, whether a group emerged with high overall desire for change showed different typicality preferences across modalities. Together, these variables captured (i) strength of desire for change, (ii) desire for atypicality and (iii) potential cross‐modal divergences.

As before, Ward's agglomerative clustering with Euclidean distance was used, and scree plots and dendograms were used to identify a three‐cluster solution. The first cluster (22%) represents people who had a strong desire to change the limb, and this desired limb was very different from a typical limb (named ‘Strong Desire for Atypicality’). The second cluster (39%) represents people who also have a desire for change, but this desired limb would match a typical limb (named ‘Desire for Typicality’). The third cluster (39%) represents people with moderate desires to change the limb, and such changes somewhat matched a typical limb, but not entirely (named ‘Desire for Some Atypicality’).

These identified clusters did not vary across modalities, and so to summarise overall desires independent of modality, we conducted an exploratory principal axis factor analysis on the 8 individual desired‐change items. Principal axis factoring extraction with oblimin rotation (Table S2) showed that a single factor accounted for 62.7% variance with a high Eigenvalue (5.02) and high Kaiser–Meyer–Olkin measure of sampling adequacy (KMO = 0.89). This supported an overall index of the extent of desired changes, so we averaged answers across the 8 items for each individual and averaged these scores to compute an overall *Desire for Change Index* (*n* = 67, Mdn = 58.13, IQR = 56.06). Similarly, factor analysis of the 8 desired‐atypicality items (Table S3) showed a single factor accounted for 92.58% variance with a high Eigenvalue (7.41) and high Kaiser–Meyer–Olkin measure of sampling adequacy (KMO = 0.92). Therefore, we averaged answers across the 8 items for each individual and averaged these scores to compute an overall *Desire For Atypicality Index* (Mdn = 50, IQR = 66.9).

To characterise the cluster profiles, we compared each cluster group according to their *Desire for Change Index* and *Desire for Atypicality Index*. These trends support the descriptions we gave to the identified clustered groups (Figure 7). The cluster profiles illustrate that individuals vary both in the extent of desired changes and in whether they would resolve their disturbances by making the limb more or less typical.

**FIGURE 7:**
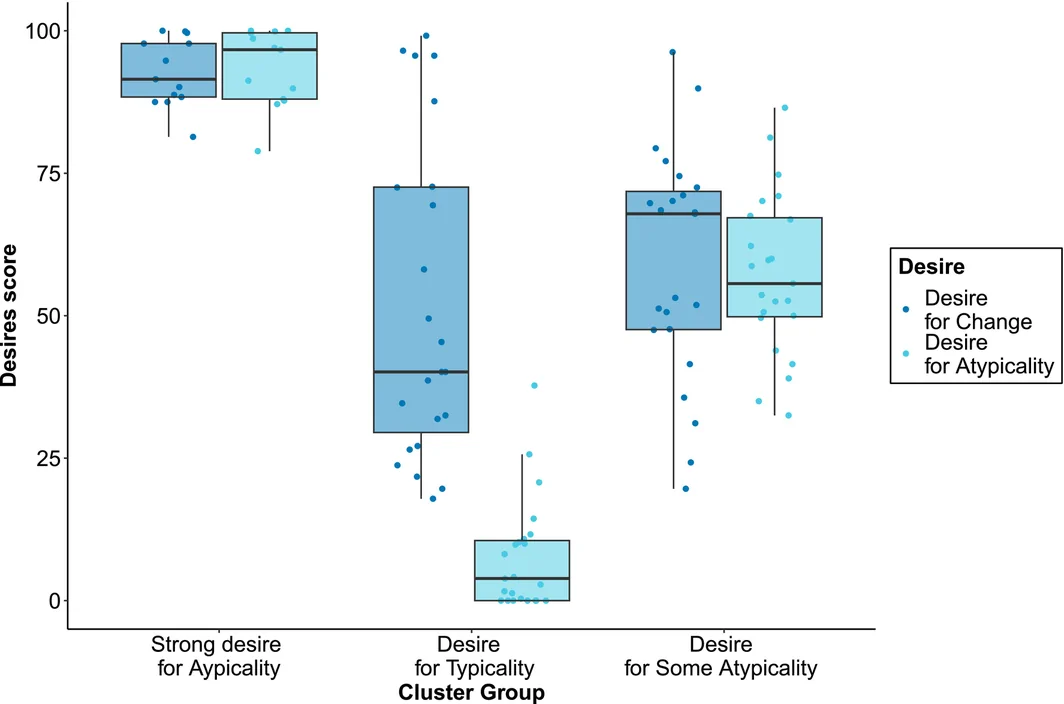
Differences in Types of Desired Changes to Body Perception in CRPS. The horizontal black line represents the median, the lower and upper hinges correspond to the first and third quartiles (the 25th and 75th percentiles), and the whiskers represent 1.5 IQR from the hinges. Dots represent individual participant data.

### 3.3 Relationship Between Perceptual Disturbances and Other Clinical Measures

#### 3.3.1 Clinical Profile of Sample

Overall, the sample presented with sensory, motor and pain experiences typically associated with CRPS. Motor difficulties were common (*n* = 52, Mdn = 76, IQR = 31.75) and had a notable impact on daily functioning (Mdn = 83.5, IQR = 17.25). CRPS Severity Scores were high (*n* = 52, Mdn = 7.5, IQR = 1), and all respondents reported allodynia or hyperalgesia and motor changes. Brief Pain Inventory scores showed that pain severity was in the moderate–severe range (*n* = 115, Mdn = 6.75, IQR = 1.75) and pain interference was substantial (*n* = 52, Mdn = 7.21, IQR = 1.89; see Shi et al. 2017 for interpreting BPI thresholds). Scores on the r‐B‐CRPS‐BPD scale indicated notable body‐perception disturbances (*n* = 115, Mdn = 26, IQR = 14.5), consistent with scores from previous reports (Ten Brink et al. 2020).

#### 3.3.2 Associations Between Body‐Perception Disturbances and Clinical Measures

We examined how clinical characteristics related to overall perceptual disturbance severity and desire for change in the affected limb (Figure 8). The *Perceptual Disturbance Index* had significant positive correlations with BPI Severity (*n* = 92, *r* = 0.390, *p* = 0.002), BPI Interference (*n* = 52, *r* = 0.49, *p* = 0.004), CRPS Severity Score (*n* = 52, *r* = 0.45, *p* = 0.011) and r‐B‐CRPS‐BPD scale Score (*n* = 92, *r* = 0.59, *p* < 0.001), indicating that greater disturbance severity corresponded with more pronounced clinical symptoms. In addition, the *Desire for Change Index* had a significant positive correlation with r‐B‐CRPS‐BPD scale Score (*n* = 67, *r* = 0.51, *p* < 0.001); however, it did not significantly correlate with the BPI Severity (*n* = 67, *r* = 0.33, *p* = 0.101), BPI Interference (*n* = 49, *r* = 0.39, *p* = 0.094) and CRPS Severity Score (*n* = 49, *r* = 0.24, *p* = 0.096) once Bonferroni corrections were applied. This suggests that desire to change limb perception relates more strongly to body‐perception disturbances themselves rather than other CRPS symptoms.

**FIGURE 8:**
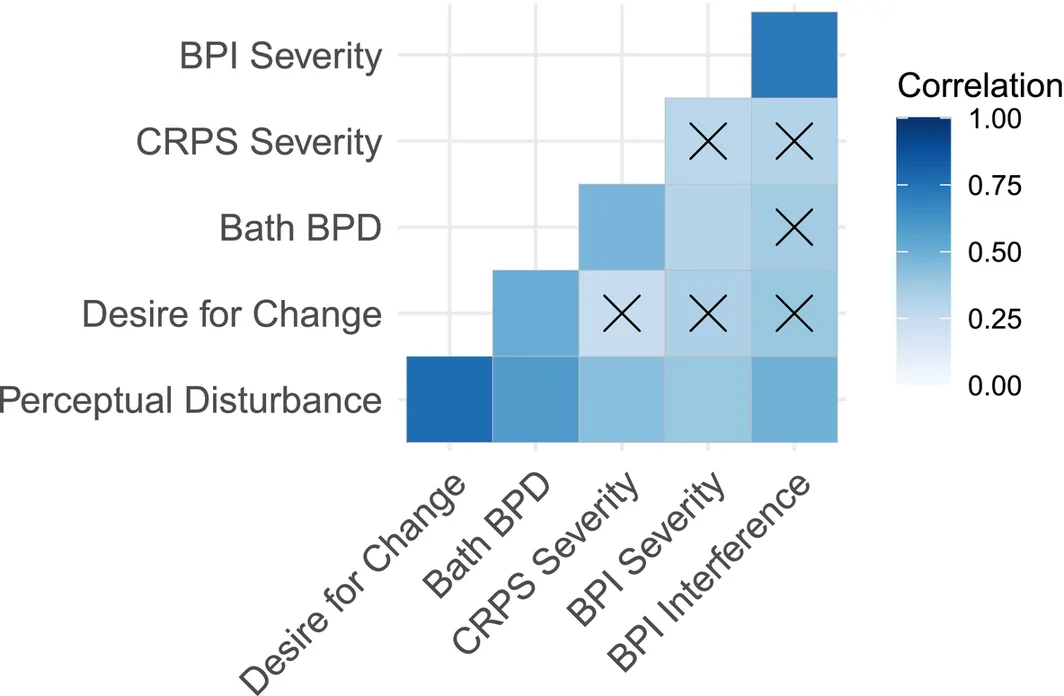
Correlation matrix to show the relationships between body perception disturbances, desired changes and other clinical measures. ‘×’ indicates non‐significant Pearson's correlation, following Bonferroni corrections. All other correlations are significant (*p*s < 0.05). BPI Severity refers to the Brief Pain Inventory Severity score. BPI interference refers to the Brief Pain Inventory Interference score. CRPS severity refers to the CRPS Severity Scale score. r‐B‐CRPS‐BPD refers to the Bath CRPS Body‐Perception Disturbance score. Perceptual Disturbance refers to our Perceptual Disturbance Index. Desire for Change refers to our Desire for Change Index.

#### 3.3.3 Clinical Patterns Across Cluster Groups

We compared the clinical characteristics of the three clusters identified according to their body‐perception disturbance patterns (Table 1). Kruskal–Wallis non‐parametric one‐way ANOVAs identified significant differences between the three cluster groups in r‐B‐CRPS‐BPD scale scores, CRPS Severity scores, BPI Interference scores and Desire for Change Indexes with small‐moderate effect sizes; however, there were no significant differences for BPI Severity scores or Desire for Atypicality Indexes (Table S4). Dwass–Steel–Critchlow–Flinger pairwise comparisons (Table S5) demonstrate that the ‘Severe Disturbances with Multimodal Agreement’ group had significantly higher r‐B‐CRPS‐BPD scale, CRPS Severity, BPI Interference and *Desire for Change Index* than the ‘*Minimal Disturbances*’ group. The only other significant difference identified was that the r‐B‐CRPS‐BPD scale score was higher in the ‘Disturbances with Multimodal Disagreement’ group compared to the ‘*Minimal Disturbances*’ group.

**TABLE 1:** CRPS clinical symptoms across body‐perception disturbance clusters.

	Severe disturbances with multimodal agreement Mdn (IQR)	Minimal disturbances with multimodal agreement Mdn (IQR)	Disturbances with multimodal disagreement Mdn (IQR)
r‐B‐CRPS‐BPD Scale	29 (10.5)^a^	18 (12)^a^, ^b^	25 (8)^b^
CRPS Severity Scale	8 (1)^a^	7 (1.5)^a^	7 (0.5)
BPI Severity	7 (1.3)	6.3 (2.5)	6 (1.5)
BPI Interference Score	8 (2)^a^	6.6 (1.7)^a^	7 (2)
Desire for Change Index	72.6 (44.2)^a^	21.7 (21.9)^a^	58.1 (48.8)
Desire for Atypicality Index	53.6 (66.9)	22.5 (51.1)	31.1 (74.5)

*Note:* Symbols show significant differences identified by the Dwass–Steel–Critchlow–Flinger tests.

^a^ Shows significant differences between the severe disturbances with multimodal agreement and the minimal disturbance groups.

^b^ Shows significant differences between the minimal disturbance and the disturbance with multimodal disagreement groups.

Next, we compared the clinical characteristics of the three cluster groups identified based on patterns in their desired changes (Table 2). Kruskal–Wallis non‐parametric one‐way ANOVAs indicated significant differences between these groups in their BPI Severity scores and *Perceptual Disturbances Index*, with small‐moderate effect sizes. However, there were no significant differences for the Bath BPD scores, CRPS Severity scores, BPI Interference scores, or *Multimodal Discrepancy Index* scores (Table S6). Dwass–Steel–Critchlow–Flinger pairwise comparisons found that the *Strong Desire for Atypicality* group had significantly higher BPI Severity scores and *Perceptual Disturbance Index* than both the ‘Desire for Typicality’ and ‘Desire for Some Atypicality’ groups (Table S7). There were no significant differences between the ‘*Desire for Typicality*’ and ‘*Desire for Some Atypicality*’ groups.

**TABLE 2:** CRPS clinical symptoms across desired change clusters.

	Strong desire for atypicality Mdn (IQR)	Desire for typicality Mdn (IQR)	Desire for some atypicality Mdn (IQR)
r‐B‐CRPS‐BPD Scale	33 (11)	25 (12)	27 (11)
CRPS Severity Scale	8 (1)	8 (1)	7 (1)
BPI severity	7.8 (1.8)^a^, ^b^	6.3 (1.1)^a^	6.8 (1.3)^b^
BPI Interference Score	8.9 (2.1)	7.1 (2.1)	7.1 (1.6)
Perceptual Disturbance Index	77.8 (28.4)^a^, ^b^	58 (36.6)^a^	65.9 (33.8)^b^
Multimodal Discrepancy Index	10 (9.3)	11 (12.1)	10 (13)

*Note:* Symbols show significant differences identified by the Dwass–Steel–Critchlow–Flinger tests.

^a^ Shows significant differences between the strong desire for atypicality and desire for typicality groups.

^b^ Shows significant differences between the strong desire for atypicality and desire for some atypicality groups.

We explored whether individuals' current perceptual profiles aligned with their desired perceptual profiles. No consistent pattern emerged (Table S8), aside from the observation that no respondents in the ‘Minimal Disturbances with Multimodal Agreement’ group were also categorised into the ‘Strong Desire for Atypicality’ group. A Fisher's exact test confirmed there was no significant pattern in the distribution across groups (*p* = 0.183).

#### 3.3.4 Subjective Impact of Perceptual Disturbances

Respondents provided subjective ratings of how their perception of their limb affected their daily life. The perceived visual appearance of the limb impacted general life satisfaction for 16% (*n* = 55), impacted general functioning for 63% (*n* = 54), and impacted specific daily activities for 91% (*n* = 55). The perceived feeling of the limb did not impact general life satisfaction (0%, *n* = 55), but did have a substantial impact on general functioning (93%, *n* = 54) and specific daily activities (100%, *n* = 55). There were no particular trends in the types of activities more or less affected, other than whether the activity predominantly used the upper or lower limb, depending on which was affected (Table S9).

Respondents anticipated some improvements in general functioning if their limb could be changed to match their desired visual appearance (Mdn = 75, IQR = 41) or feeling (Mdn = 73, IQR = 52). They also reported expected improvement across the specific activities (average of expected improvement score for each activity) if their limb could be changed to match their desired visual appearance (Mdn = 58.9, IQR = 65.29) or feeling (Mdn = 60.6, IQR = 46.7), though again with much variability (Table S10).

## 4 Discussion

We aimed to characterise how body perception is distorted in CRPS, how people with CRPS want their affected limb to be, and the relationships between these phenomena. Disturbances varied in the extent to which the affected limb was distorted from how it typically should be and the discrepancy between the limb's visual appearance and feel. Most people reported a desired limb perception that was not like a typical limb.

### 4.1 Characterising Body‐Perception Disturbances

Consistent with previous studies (Förderreuther et al. 2004; Lewis et al. 2007; Lewis and McCabe 2010; McCabe et al. 2005), disturbances in affected limb perception were widespread. Distortions in size, shape, definition and position were strongly related, and most individuals experienced similar magnitudes of visual and felt distortions. However, the severity of distortions varied, with over half (58%) reporting pronounced distortions compared to a typical limb, despite visual and felt perceptions corresponding. The similarity between visual and felt disturbances is rarely clarified in the literature, or disturbances are defined only as differences between modalities, as in the r‐B‐CRPS‐BPD scale (Halicka et al. 2020; Lewis et al. 2007; Lewis and McCabe 2010; Ten Brink et al. 2020). However, by explicitly asking about differences between the current limb perception and the expected state of a typical limb, we could identify perceptual disturbances occurring in both modalities. This distinction proved useful in interpreting the clinical profiles of different cluster groups. Of the two groups with multimodal agreement, the group that perceived greater distortions from a typical limb showed higher CRPS severity, BPI interference, r‐B‐CRPS‐BPD scale scores, and stronger desires to change their limb. As such, the greater the divergence of the affected limb's perception from a typical limb, the more severe the CRPS symptoms are.

Deviations between limb perception and a typical limb may reflect conflicts between the limb's physical, remembered, or expected state. Some internal body representations are informed by past experiences or knowledge, creating a stable idea of what the body should be like (‘offline’ body representation) (Carruthers 2008). This may conflict with ‘online’ representations stemming from moment‐to‐moment bodily information, which could explain the reported perceptual distortions, especially if individuals are consciously aware of this conflict (Brun et al. 2019; Lewis et al. 2021; Pitron et al. 2018; Tsay et al. 2015). For example, one theory explaining CRPS body‐perception disturbances posits that stable representations of how the limb ‘should be’ are not updated following pain and pain behaviours, creating a mismatch between the body's expected and actual state (Brun et al. 2021; Harris 1999; McCabe and Blake 2007). Future work could investigate the correspondence between the limb's physical, perceived and expected state to clarify how best to define perceptual disturbances in CRPS.

Despite generally high correspondence between visual and felt disturbances, a small group of respondents showed discrepancies between these modalities, mirroring previous findings (Brun et al. 2021; Harris 1999) and questions in r‐B‐CRPS‐BPD scale (Lewis and McCabe 2010; Ten Brink et al. 2020), which ask about conflicts between visual and felt limb perceptions. Such discrepancies resemble the dissociation between visual and felt perceptions of the body illustrated in healthy individuals (Longo 2015), though the reasons for multimodal disagreements in CRPS arising for only some individuals are unclear. Furthermore, our analyses revealed no clear evidence that the multimodal disagreement was related to clinical measures, although this could be due to the lower sample size for this group.

Altogether, our findings highlight distinctions in the level of multimodal agreement and degree of distortion from a typical limb in CRPS, which may reflect changes to separable online and offline body representations.

### 4.2 Characterising Desired Changes to Affected Limb

Another novel insight was that we could distinguish groups by their desire to change their limb perception and the extent to which their desired perception would differ from a typical limb. Cluster analyses indicated that one group of respondents (39%) strongly desired changes in the perceived visual appearance and feeling of their limb, such that it would resemble a typical limb; this aligns with treatments aiming to normalise body perception by matching sensory information to a typical limb (Boesch et al. 2016; McCabe et al. 2008; Sayegh et al. 2013).

Contrastingly, a second group (39%) had a more moderate desire for change, with their desired limb perception being somewhat atypical relative to a typical limb. A third group (22%) had a stronger desire for change, but desired markedly more atypical changes than the other groups. This group's lack of desire for normalisation is important, as many rehabilitation strategies aim to normalise limb perception (Arcos‐Holzinger et al. 2023; Boesch et al. 2016; Lewis and McCabe 2010). Furthermore, they exhibited more severe clinical presentation, with higher Perceptual Disturbances Indexes, BPI Severity and Desire for Change Indexes. Whilst initially surprising, wanting a limb's perceived appearance or feel to be somewhat atypical could be an attempt to resolve conflicting body representations and achieve perceptual coherence, rather than imposing a normal appearance that still conflicts with internal body maps, for instance. Our cluster analyses show that individuals with CRPS have distinct, subjective ideas of how they wish their limb perception could change, suggesting that categorising these desired perceptions could inform treatment decisions and indicate when normalisation may not improve perceptual experiences.

Further exploration of desired limb perception in CRPS could be valuable. Conscious body perception is subjective, influenced by various factors contributing to higher order neural representations of the body (Viceconti et al. 2020). Therefore, resolving body‐perception conflicts may require consideration of individual perceptual experiences. Recently, Lewis et al. (2021) showed that exposing people with CRPS to a virtual reality hand that matched their desired appearance led to lower pain intensity and r‐B‐CRPS‐BPD scale scores. They suggested that embodying the desired limb helped resolve central body representation conflicts and restore subjective body‐perception coherency (Lewis et al. 2021). Our study extends this finding by showing that people with CRPS differ meaningfully in their desired changes in limb perception, that these desires do not always correspond to limb normalisation, and that these patterns are associated with differences in wider CRPS symptoms. This suggests that understanding the nature and motivations underlying desired changes could help identify more individualised rehabilitation targets.

### 4.3 Functional Impact

Almost all respondents reported that the appearance or feeling of their affected limb impairs at least one daily activity, with the majority reporting impairments to general functioning. This builds on prior research linking body‐perception disturbances to motor functioning in CRPS (Bultitude and Rafal 2010; Lewis and Schweinhardt 2012), by demonstrating that people with CRPS believe their disturbed limb perception has functional consequences. Some respondents felt their functioning would improve if they could change their affected limb as desired, but this varied, suggesting motivations for desired changes beyond functional benefits. These results emphasise the importance of understanding how perceptual disturbances impact limb function.

### 4.4 Limitations

Online delivery of our survey provided a large and comprehensive dataset to explore patterns within a heterogeneous phenomenon, but had several limitations. First, we cannot ensure that reported visual distortions are purely perceptual rather than actual physical changes (e.g., swelling). Only 47% reported their limb's perceived appearance matched its physical state, though this may simply reflect an inaccurate perception of the limb. Second, respondents might have misunderstood some questions. For example, discrepancies between the categorical and scale questions about desired limb appearance might stem from confusion over the complex categorical options, though we attempted to mitigate this by piloting the questionnaire. Also, our measurements of body‐perception disturbances01‐B‐CRPS‐BPD scale (Ten Brink et al. 2020; Lewis and McCabe 2010), supporting the validity of our bespoke questions. Our results provide a useful starting point for exploring current and desired body perception in more detail.

### 4.5 Implications

Our findings highlight the conceptual and clinical importance of explicitly defining body‐perception disturbances in CRPS. Previous research is ambiguous about whether these disturbances reflect differences between seen and felt perceptions, deviations from a typical limb or differences in the imagined state (mental representation) of the limb. Our identification of separate cluster groups based on distortion severity and multimodal agreement suggests these are distinct ways limb perception can vary in CRPS.

Moreover, understanding patients' desired limb perceptions holds value. Pain severity varied significantly with desired limb perception but not current body‐perception disturbances, illustrating the clinical relevance of desired perceptions. Since most participants desired a limb different from a typical limb, common treatments aiming to normalise the limb may not suit all CRPS profiles. For example, normalising visual appearance may not resolve perceptual conflict if typical visual cues are already present but not coherently integrated with felt or other body representations. Comparing current and desired perceptions in future research could reveal whether patients seek normalisation, greater congruence between representations, or changes in the opposite direction. This would help identify more individualised treatment targets that accommodate patients' current perceptions and attitudes towards their own limb perception.

## 5 Conclusions

The insights from this study challenge current conceptualisations of body‐perception disturbances in CRPS. First, body‐perception disturbances are often characterised as a single construct; however, our study reveals three distinct clusters differentiated by the severity of distortions from a typical limb and by the conflict between visual and felt disturbance severity. Second, our findings challenge assumptions that clinical practice should aim to normalise limb appearance to improve body perception in CRPS, as many individuals would make atypical alterations to their limb perception. Future research should explore alternative ways to achieve body‐perception coherence that reflect the subjective and varied nature of body perception in CRPS.

## Author Contributions

C.E.N. is primarily responsible for the inception and design, with contributions from J.H.B., G.B. and C.L. C.E.N. collected the data and conducted the analysis, working closely with J.H.B. to make decisions throughout the exploratory analysis. J.H.B. and G.B. critically examined the analysis. C.E.N. drafted the manuscript, which was edited and redrafted by J.H.B. and G.B., and further reviewed by C.L. All authors have approved the final version of the manuscript and agree to be accountable for all aspects of the work.

## Funding

This work was funded by the UKRI Centre for the Analysis of Motion, Entertainment Research and Applications (CAMERA 2.0), University of Bath, as part of a PhD studentship.

## Conflicts of Interest

J.H.B. is Co‐Chair of the IASP special interest group for CRPS; has received fees for speaking about CRPS paid by Theoria Congresos, at a conference organised by Hospital Intermutual de Levante; and has received research funding from the Reflex Sympathetic Dystrophy Syndrome Association, the Netherlands Organisation for Scientific Research and the Experimental Psychological Society. There are no other conflicts of interest to declare.

## Supporting information


**Table S1:** Exploratory factor analysis of perceptual disturbance questions.
**Table S2:** Exploratory factor analysis of desire for change questions.
**Table S3:** Exploratory factor analysis of desire for atypicality questions.
**Table S4:** Kruskal–Wallis non‐parametric one‐way ANOVA to identify whether CRPS clinical symptoms differ across body perception disturbance clusters.
**Table S5:** Dwass–Steel–Critchlow–Flinger pairwise comparisons of clinical symptoms across body perception disturbance clusters.
**Table S6:** Kruskal–Wallis non‐parametric one‐way ANOVA to identify whether CRPS clinical symptoms differ across desired change clusters.
**Table S7:** Dwass–Steel–Critchlow–Flinger pairwise comparisons of clinical symptoms across desired change clusters.
**Table S8:** Percentage of people belonging to each pair of current perception and desired cluster groups.
**Table S9:** Extent to which visual and felt limb perception impacts specific daily activities.
**Table S10:** Extent to which respondents believe that desired changes would improve their ability to perform daily tasks.
**Table S11:** Categorical responses for whether desired limb resembles a typical limb or current limb perception.
