# Supplementary material for: Variations in Current and Desired Limb Perception in Complex Regional Pain Syndrome
Source: Eur J Pain. 2026 Oct 9;30(9):e70401. doi: 10.1002/ejp.70401 (PMC13651783; doi:10.1002/ejp.70401)
Supplement: Supplementary file 1 [file EJP-30-0-s001.docx]

**Variations in current and desired limb perception in complex regional pain syndrome**

**Supplementary Materials**

***Table S1.***

*Exploratory factor analysis of perceptual disturbance questions*

| **Item** | **Factor Loading** |
| --- | --- |
| Visual Appearance – Size | 0.89 |
| Feeling – Shape | 0.86 |
| Feeling – Size | 0.85 |
| Visual Appearance – Definition | 0.85 |
| Feeling – Definition | 0.84 |
| Visual Appearance – Shape | 0.83 |
| Visual Appearance – Position | 0.81 |
| Feeling – Position | 0.79 |

*Note.* Factor loadings from a principal axis factoring extraction method with oblimin rotation.

***Table S2.***

*Exploratory Factor Analysis of Desire for Change questions*

| **Item** | **Factor Loading** |
| --- | --- |
| Visual Changes – Shape | 0.88 |
| Visual Changes – Size | 0.85 |
| Visual Changes – Definition | 0.84 |
| Felt Changes – Definition | 0.80 |
| Felt Changes – Shape | 0.75 |
| Visual Changes – Position | 0.74 |
| Felt Changes – Position | 0.74 |
| Felt Changes – Size | 0.73 |

*Note.* Factor loadings from a principal axis factoring extraction method with oblimin rotation.

***Table S3.***

*Exploratory Factor Analysis of Desire for Atypicality questions*

| **Item** | **Factor Loading** |
| --- | --- |
| Felt Desired Atypicality - Size | 0.99 |
| Felt Desired Atypicality - Shape | 0.98 |
| Felt Desired Atypicality - Position | 0.98 |
| Felt Desired Atypicality - Definition | 0.97 |
| Visual Desired Atypicality - Shape | 0.96 |
| Visual Desired Atypicality - Size | 0.95 |
| Visual Desired Atypicality - Position | 0.94 |
| Visual Desired Atypicality - Definition | 0.93 |

*Note.* Factor loadings from a principal axis factoring extraction method with oblimin rotation.

**Table S4.**

*Kruskal-Wallis non-parametric One-Way ANOVA to identify whether CRPS clinical symptoms differ across body perception disturbance clusters.*

|  | $\mathcal{X}^{2}$ *(all 2 DoF)* | *p* | $\varepsilon^{2}$ |
| --- | --- | --- | --- |
| **r-B-CRPS-BPD**  *(n = 92)* | 30.07 | <.001 | 0.33 |
| **CRPS Severity**  *(n = 52)* | 11.38 | .003 | 0.22 |
| BPI Severity  *(n = 92)* | 5.10 | .078 | 0.06 |
| **BPI Interference Score**  *(n = 52)* | 7.85 | .020 | 0.15 |
| **Desire for Change Index**  *(n = 67)* | 23 | <.001 | 0.35 |
| Desire for Atypicality Index  *(n = 59)* | 3.62 | .164 | 0.06 |

**Table S5.**

*Dwass-Steel-Critchlow-Flinger pairwise comparisons of clinical symptoms across body perception disturbance clusters.*

|  | Severe disturbances vs minimal disturbances | Severe disturbance vs multimodal disagreement | Minimal disturbances vs multimodal disagreement |
| --- | --- | --- | --- |
| r-B-CRPS-BPD | *W* = 7.58, *p* <.001* | *W* = 2.38, *p* = .212 | *W* = 3.33, *p* = .048* |
| CRPS Severity | *W* = 4.54, *p* = .004* | *W* = 2.49, *p* = .183 | *W* = 0.70, *p* = .875 |
| BPI Interference | *W* = 3.92, *p* = .016* | *W* = 1.35, *p* = .604 | *W* = 0.80, *p* = .838 |
| Desire for Change Index | *W* = 6.58, *p* <.001* | *W* = 2.20, *p* = .264 | *W* = 3.17, *p* = .065 |

**Table S6.**

*Kruskal-Wallis non-parametric One-Way ANOVA to identify whether CRPS clinical symptoms differ across desired change clusters.*

|  | $\mathcal{X}^{2}$ *(all 2 DoF)* | *p* | $\varepsilon^{2}$ |
| --- | --- | --- | --- |
| r-B-CRPS-BPD  *(n = 59)* | 3.61 | .164 | 0.06 |
| CRPS Severity  *(n = 43)* | 1.53 | .466 | 0.04 |
| **BPI Severity**  *(n = 59)* | 11.94 | .003 | 0.21 |
| BPI Interference Score  *(n = 43)* | 5.24 | .073 | 0.12 |
| **Perceptual Disturbance Index**  *(n = 59)* | 9.67 | .008 | 0.17 |
| Multimodal Discrepancy Index  *(n = 59)* | 1.1 | .576 | 0.02 |

**Table S7.**

*Dwass-Steel-Critchlow-Flinger pairwise comparisons of clinical symptoms across desired change clusters.*

|  | Strong desire for atypicality vs desire for typicality | Strong desire for atypicality vs desire for some atypicality | Desire for typicality vs desire for some atypicality |
| --- | --- | --- | --- |
| BPI Severity | *W* = 4.622, *p* = .003* | *W* = 3.43, *p* = .040* | *W* = 2.12, *p* = .292 |
| Perceptual Disturbance Index | *W* = 4.08, *p* = .011* | *W* = 3.84, *p* = .018* | *W* = 0.03, *p* = .100 |

***Table S8.***

*Percentage of people belonging to each pair of current perception and desired cluster groups*

|  | Severe Disturbance with Multimodal Agreement  (n = 41) | Minimal Disturbances  (n = 12) | Disturbances with Multimodal Disagreement  (n = 6) |
| --- | --- | --- | --- |
| Strong Desire for Atypicality | 27% | 0% | 33% |
| Desire for Typicality | 34% | 50% | 50% |
| Desire for Some Atypicality | 39% | 50% | 17% |

*Note.* There were 59 people who were assigned both a current perception cluster group and a desired perception cluster group. The table reports the percentage of people from each perceptual disturbance cluster that appear in each of the desired changes clusters.

***Table S9.***

*Extent to which visual and felt limb perception impacts specific daily activities.*

|  | **Impairment related to limb’s visual appearance**  Freq. of ‘yes’ | | **Impairment related to limb’s feeling**  Freq. of ‘yes’ | |
| --- | --- | --- | --- | --- |
|  | **Upper** | **Lower** | **Upper** | **Lower** |
| **Grasping a mug** | 60% | 0% | 85% | 0% |
| **Lifting a cup** | 75% | 0% | 90% | 0% |
| **Writing** | 55% | 0% | 65% | 0% |
| **Typing** | 70% | 11% | 85% | 9% |
| **Brushing teeth** | 55% | 3% | 75% | 9% |
| **Walking up stairs** | 15% | 80% | 25% | 91% |
| **Walking for ten minutes** | 15% | 83% | 25% | 94% |
| **Getting dressed** | 75% | 77% | 95% | 80% |
| **Showering/ bathing** | 75% | 89% | 85% | 94% |
| **Preparing food** | 80% | 66% | 90% | 70% |
| **Tying shoelaces** | 80% | 77% | 90% | 91% |
| **Bending to pick up** | 50% | 77% | 60% | 86% |

*Note.* ‘Upper’ refers to those who reported impairments on each activity, of the 20 people with upper-limb CRPS who answered these questions. ‘Lower’ refers to those who reported impairments, of the 35 people with lower-limb CRPS who answered these questions.

***Table S10.***

*Extent to which respondents believe that desired changes would improve their ability to perform daily tasks.*

|  | **Expected improvement from desired visual changes**  Mdn (IQR) | | **Expected improvement from desired felt changes**  Mdn (IQR) | |
| --- | --- | --- | --- | --- |
|  | **Upper** | **Lower** | **Upper** | **Lower** |
| **Grasping a mug** | 67.5 (55) | N/A | 68 (48.8) | N/A |
| **Lifting a cup** | 65 (59) | N/A | 72 (68) | N/A |
| **Writing** | 63 (88) | N/A | 49.5 (61.5) | N/A |
| **Typing** | 79 (88.5) | 21 (28) | 55 (68.5) | 55 (68.5) |
| **Brushing teeth** | 45 (54.8) | 100 (0) | 63.5 (49.3) | 61.5 (38.5) |
| **Walking up stairs** | 83.5 (16.5) | 69 (66.5) | 33.5 (50.3) | 50 (64.5) |
| **Walking for ten minutes** | 49 (0) | 70 (73) | 42.5 (17.5) | 55 (67) |
| **Getting dressed** | 52 (69.5) | 69 (66) | 60 (44.5) | 51 (53.5) |
| **Showering/ bathing** | 43 (69) | 60 (62) | 60 (45) | 51 (53.5) |
| **Preparing food** | 63.5 (80.3) | 55 (74) | 73 (67) | 50.5 (73) |
| **Tying shoelaces** | 47 (88) | 81 (81) | 49 (76) | 57.5 (43.3) |
| **Bending to pick up** | 46 (61.5) | 72 (74.5) | 30 (54.5) | 61.5 (53.5) |

*Note.* Only those who answered ‘yes’ to their affected limb perception impacting their functioning on these tasks (I.e. Table S4), were asked about their expectations for improvements if their desired changes were made.

**5. Additional Exploratory Categorical Questions**

In order to understand whether people desired their limb to resemble a typical limb, or whether they wanted vision and feeling to match (even if these were distorted), we asked some questions in addition to those reported in the main report. We asked respondents to indicate, if they made the changes to the way their limb looks, how would the visual appearance of their limb compare to the way they currently imagine or think about their limb. They had to select one of the following answers: it would be identical, it would be somewhat different, it would be very different or unsure. They were also asked a similar question about whether the limb would look like a typical limb, with the same response choices. These two questions were then repeated regarding changes to the feeling of the limb: for example, if changes were made, how would the feeling of their limb compare to how the limb currently looks.

As shown in Table S11, categorical responses about whether the desired limb would resemble a typical limb did not fully mirror the results reported in section 3.2.1 from the continuous ratings. In the scale questions, most respondents indicated some difference from a typical limb, whereas the categorical responses produced a higher proportion reporting the desired limb would be identical to a typical limb. We also saw variability in the extent to which desired changes were intended to reduce discrepancies between the visual and felt perceptions of the limb. No consistent patterns emerged across the categorical responses. Taken together, these findings indicate that participants’ desired limb perceptions are more nuanced than a simple typical/atypical categorical distinction, and continuous scales may afford a more sensitive measure of these preferences. Qualitative analyses, reported separately, will further illuminate these nuanced desires.

***Table S11.***

*Categorical responses for whether desired limb resembles a typical limb or current limb perception.*

| **The question** | **n** | **Identical** | **Somewhat different** | **Very different** | **Unsure** |
| --- | --- | --- | --- | --- | --- |
| How would the visual appearance of the desired limb compare to a typical limb? | 75 | 73% | 12% | 4% | 11% |
| How would the visual appearance of the desired limb compare to the way you currently imagine or think about your limb? | 75 | 67% | 14% | 4% | 14% |
| How would the feeling of the desired limb compare to a typical limb? | 69 | 21% | 19% | 39% | 21% |
| How would the feeling of the desired limb compare to the way your limb currently looks to you? | 69 | 25*%* | 23% | 36% | 16% |
